# Aorto-mesenteric space reduction in women with anorexia nervosa: retrospective audit and analysis

**DOI:** 10.1186/s40337-026-01566-1

**Published:** 2026-03-06

**Authors:** Léo Taieb, Yacine Meziane, Yohann Renard, Marion Barrois, Cyril Cosse, Christine Hoeffel, Guillaume Cadiot, Eric Bertin

**Affiliations:** 1 Department of Endocrinology, Diabetes and Nutrition, Reims University Hospital, 45 rue Cognacq Jay, 51100 Reims, France; 2 Department of Diagnostic and Interventional Imaging in Adults, Reims University Hospital, Reims, France; 3 LICIIS, University of Reims Champagne-Ardenne, Reims, France; 4 Emergency Department, Louis Mourier University Hospital, Colombes, France; 5 CReSTIC, UR 3804, University of Reims Champagne-Ardenne, Reims, France; 6 Department of Gastroenterology, Reims University Hospital, Reims, France; 7 Performance, Health, Metrology, Society Laboratory, UR 7507, University of Reims Champagne-Ardenne, Reims, France

**Keywords:** Anorexia nervosa, Undernutrition, Superior mesenteric artery syndrome, Nutcracker syndrome, Dyspepsia

## Abstract

**Background and aims:**

Superior mesenteric artery syndrome (SMAS) is a rare condition favored by weight loss, with nonspecific digestive complaints that may hinder weight recovery in anorexia nervosa. This study aimed to examine the radiological features of aorto-mesenteric (A-M) space reduction in anorexia nervosa and their association with digestive complaints.

**Methods:**

Female patients with anorexia nervosa and a history of computerized tomography angiography for digestive complaints were included. Clinical data were retrospectively collected and computerized tomography scans were reviewed by an independent, experienced radiologist to identify signs of SMAS and of Nutcracker syndrome. Adipose tissue surfaces were also calculated from the scans. Some patients were reassessed after nutritional recovery.

**Results:**

On the 51 female patients included (mean age 27.7 ± 12.3 years) from a cohort of 202 female anorexia nervosa patients, 48 met radiological criteria for SMAS (A-M angle < 25° or distance ≤ 8 mm). A duodenal dilation was present in 35 patients (68.6%) and a left renal vein dilation in 39.2%. The type of digestive complaints did not differ significantly between patients with and without duodenal dilation, although gastroesophageal reflux approached statistical significance (*p* = 0.06). Body mass index and visceral adipose tissue did not correlate significantly with A-M measurements. Following nutritional recovery in ten patients, there was a significant increase in A-M measurements and a decrease in both duodenal dilation and digestive symptoms.

**Conclusion:**

Radiological features of A-M space reduction are common in anorexia nervosa. Left renal vein compression and its upstream dilation, as well as left dilated ovarian vein and pelvic varicose veins, are frequently associated with radiological signs of SMAS. Nutritional support alleviates digestive complaints related to SMAS.

**Supplementary Information:**

The online version contains supplementary material available at 10.1186/s40337-026-01566-1.

## Abstract (plain language summary)

This study examined how the space between the body’s main artery (the aorta) and the artery that supplies blood to the intestines (the superior mesenteric artery) can become narrower in people with anorexia nervosa in relation with body weight decrease. This narrowing happens where the first part of the small intestine (the duodenum) passes between the two arteries. It may contribute to digestive problems that are common in anorexia nervosa. The results showed that 94.1% of patients had a significantly narrowed space between these arteries (a condition sometimes called superior mesenteric artery syndrome), and 68.6% had an enlarged duodenum. Among digestive symptoms, acid reflux (heartburn) was the most strongly linked to this enlargement. After nutritional recovery, the space between the arteries widened again, and both the duodenal enlargement and digestive symptoms improved. These findings are important because they show that this narrowing is much more common in anorexia nervosa than previously thought. It may help explain why so many patients experience digestive issues. This insight could guide clinicians in selecting the most appropriate nutritional support strategies, which are a critical component of anorexia nervosa treatment.

**Supplementary Information:**

The online version contains supplementary material available at 10.1186/s40337-026-01566-1.

## Introduction

Superior mesenteric artery syndrome (SMAS), also named Wilkie’s syndrome or Cast syndrome, was firstly described by Carl Von Rotikansky in 1861. The syndrome is more frequent in females (sex ratio 2:1), with an estimated prevalence from 0.013% to 0.3%, depending on clinician and radiologist awareness of the syndrome and its diagnostic criteria [1, 2].

Clinical manifestations of SMAS include nonspecific digestive symptoms such as early satiety, postprandial pain or discomfort, nausea and bilious emesis (often postprandial), bloating, eructation and esophageal reflux, anorexia, and weight loss [3, 4]. These symptoms typically develop insidiously, leading to diagnostic delay [5–7]. In some cases, however, SMAS may present as acute upper intestinal ileus [8].

SMAS results from vascular compression of the third portion of the duodenum within the space bounded by the superior mesenteric artery (which originates from the aorta at an acute downward angle) and the aorta. Any condition that narrows the aorto-mesenteric (A-M) angle predisposes to SMAS. The most common underlying mechanism is a reduction in the mesenteric fat pad [9] resulting from various weight loss situations such as chronic illness, malignancy, eating disorder [10]. Less frequently, SMAS is associated with surgical treatment for scoliosis, exaggerated lumbar lordosis, external compression (e.g., cast treatment), a short and/or hypertrophic ligament of Treitz [11], or a low origin of the superior mesenteric artery [12].

Diagnosis of SMAS, once clinically suspected, can be confirmed using upper gastrointestinal radiography, abdominal computed tomography (CT) angiography, or magnetic resonance angiography. Diagnostic criteria include duodenal dilation (with or without gastric dilation), abrupt compression of the third duodenal segment (D3) behind the superior mesenteric artery, and/or a delayed barium transit through the duodenum, all of which indicate functional impairment due to D3 compression between the two arteries [13].

CT angiography is considered the gold standard for assessing the A-M angle and distance [4]. An A-M angle less than 25° or an A-M distance of 8 mm or less are the usual diagnostic thresholds for SMAS on CT angiography [14]. Previous angiographic studies of SMAS cases have reported A-M angle of 6–22° and A-M distance of 2–8 mm [15]. Some authors suggest that A-M distance may be a more critical factor than A-M angle [15, 16].

Another rare entity, the nutcracker syndrome (NS), involves compression of the left renal vein between the superior mesenteric artery and the aorta, leading to symptoms of renal congestion. Macroscopic and microscopic hematuria is the most common presentation, but NS has also been associated with proteinuria, flank and pelvic pain and gonadal varices [17, 18]. Headaches have also been reported in this syndrome and may be the sole clinical manifestation [19].

Although SMAS and NS likely share a common anatomical etiology, their co-occurence has been documented in only a few case-reports [20]. Diagnosis of NS requires a high index of clinical suspicion and represents a diagnosis of exclusion. CT angiography, magnetic resonance imaging (MRI), and Doppler ultrasound are all reasonable imaging studies for suspected NS, but invasive assessment with catheter venography and pressure measurement may be necessary to confirm that the symptoms are due to left vein compression [21]. CT may reveal compression of the left renal vein, gonadal vein distension, and pelvic congestion.

While SMAS is often unrecognized and undiagnosed, it mainly manifests as a benign duodenal stasis that can be safely managed with conservative measures aimed at correcting weight loss [3, 6]. In severe or refractory cases, however, surgical intervention with duodenojejunostomy may be required [13]. One case report documented improvement in CT findings following body weight recovery through nutritional therapy [22], but the available data in the literature are scarce and do not allow determination of the body mass index required to resolve clinical symptoms or normalize A-M measurements.

SMAS has already been reported in weight loss due to anorexia nervosa (AN) regardless of subtype: restrictive (AN-R) or binge eating/purging (AN-B/P). However, studies examining SMAS in AN have been limited to case reports, with the largest study involving only eight AN patients with digestive complaints [23].

Furthermore, most of AN patients report digestive discomfort or abdominal distension, including postprandial fullness, epigastric pain, dysphagia, nausea, bloating and constipation [24]. The origin of these symptoms appears multifactorial and complex, potentially involving visceral hypersensitivity, visceral conditioning, altered neuroplasticity in response to visceral pain, or anxiety symptoms [25]. Recent evidence also suggests that gastric distension may contribute to digestive complaints present in AN [8]. Thus, the clinical signs of SMAS can mimic dyspepsia seen in AN [13] potentially exacerbating fear and avoidance of food and further complicating nutritional recovery [26].

There is a paucity of studies on the relationships between digestive disorders and SMAS in AN, given the various confounding factors. Moreover, SMAS is often underdiagnosed in AN patients [27]. The aim of the present study is to examine radiological features of A-M space reduction in AN and their association with digestive complaints.

## Methods

### Study design

This study was conducted as a retrospective chart review, and the scan images were re-analyzed by an experienced radiologist.

### Outcomes

The primary outcomes were the frequency of radiological criteria for SMAS and NS in patients with AN and digestive symptoms. Secondary outcomes included: the identification of clinical features (including type of digestive complaints) and CT adipose tissue parameters associated with radiological criteria for SMAS; the impact of weight gain following nutritional support on radiological criteria for SMAS and NS, adipose tissue and digestive complaints.

### Population

Subjects were suitable for inclusion if they were female, diagnosed with AN according to DSM-5 criteria, aged over 16 years at initial assessment, and managed at the acute center for nutrition diseases, University Hospital of Reims, France, between January 2019 to December 2024. Patients who underwent abdominal CT angiography to detect possible SMAS were compared with AN patients who did not undergo scanning to rule out this diagnosis. Exclusion criteria were diabetes mellitus, digestive pathology potentially altering digestive or abdominal anatomy (e.g. Crohn’s, cancer, history of supra-umbilical abdominal surgery, pancreatitis sequelae), and an occlusive episode at the time of CT angiography or in the month preceding inclusion.

### Clinical data

Clinical and anthropometric characteristics were extracted from the medical records: age, height, minimum and maximum body weight, body weight at the time of CT, corresponding BMIs, AN subtype, duration of AN at the time of CT, and other potential causes of abdominal symptoms (e.g., hyperlaxity particularly Ehler-Danlos syndrome, irritable bowel syndrome).

AN subtype was determined based on the presence of vomiting and/or binge eating from the onset of restrictive behaviour until data extraction. The AN group was divided into 2 sub-groups, according to DSM-5 criteria : AN- binge/purge subtype (AN-B/P), and AN-restricting subtype (AN-R).

Abdominal complaints present at the time of CT and potentially related to SMAS were recorded: dyspepsia, early satiety, postprandial pain or discomfort, nausea and postprandial emesis, bloating, reflux and other symptoms like diffuse, left hypochondrium or peri-umbilical abdominal pain, transit disorders. Complaints suggestive of NS were pelvic pain, lower back pain, left abdominal quadrant pain (left hypochondrium, left lumbar region, left iliac region), and headache.

### Abdominal CT angiography measurements

The presence of an abdominal CT angiography and its location (inside or outside our hospital) and upper gastro-intestinal radiography were recorded. All CT scans (inpatient and outpatient) were collected via the radiological department software and then reviewed by an independent experienced radiologist, to assess for :

- Signs of SMAS: A-M angle < 25 degrees, A-M distance ≤ 8 mm, compression of the third portion of the duodenum (D3) between aorta and superior mesenteric artery, duodenal dilation,
- Position of D3 relative to vertebrae to detect a possible low duodenum level, thus avoiding arterial compression despite radiological signs of SMAS,
- Signs of NS: stenosis of the left renal vein at the level of vascular compression, dilation of the left renal vein upstream the compression, and dilation of the left ovarian vein and/or pelvic varices (see Supplementary Fig. 1S).

For A-M angle measurements, a line was drawn between the root of the superior mesenteric artery and the aorta and internal side of each artery. Angles were obtained by manual tracing, with degrees automatically calculated. The A-M distance was measured as the maximum distance between the anterior margin of the aorta and the posterior aspect of the superior mesenteric artery at the level where D3 crossed on the axial image (see Supplementary Fig. 2S) [28].

Subcutaneous adipose tissue (SAT) and visceral adipose tissue (VAT) were measured at the lumbar L4 level on abdominal CT scans. Total skeletal muscle mass (SMM) and psoas muscle mass (PMM) were measured at lumbar L3 level. It was a semi-automatic method on a dedicated post-treatment station by a single trained operator to the measurements. Structure were manually outlined and the software automatically calculated the surface area of muscle or fat, based on the number of pixels with attenuation values comprised between − 190 and − 30 Hounsfield Units (HU) for fat, and between − 29 to + 150 HU for skeletal muscle (see Supplementary Fig. 3S) [29, 30]. Skeletal muscle index (SMI) and Psoas index (PI) were then calculated using to the following formula : SMI = SMM/body height squared; PI : PMM/body height squared.

Two additional diameters were measured on CT scans : sagittal abdominal diameter on the the L3 slice, with the cursor extending from anterior to posterior skin through the center of the abdomen, and prevertebral abdominal diameter, extending from anterior skin to anterior wall of L3, in the same direction (see Supplementary Fig. 4S). All measurements were recorded to the nearest 0.1 cm by the same operator [31].

### Statistical analyses

Quantitative variables are expressed as mean ± standard deviation and as median [minimum - maximum], and compared using Student’s t-test after verifying the conditions for application. Qualitative variables are presented as counts (percentages) and compared using the Chi-squared test with Yates’ correction as needed.

For patients with two CT scans, paired series tests were applied (paired t-test for quantitative variables; McNemar’s Chi-squared test for qualitative variables).

Correlations with distances and angles were assessed using Pearson’s correlation test and are presented with the correlation coefficient (r) and its associated p-value. A positive correlation indicates that both parameters change in the same direction, and negative correlation indicates they change in opposite directions.

The significance threshold was set at 0,05. Statistical analyses were performed using SPSS software, Version 29.0.2.0 (IBM Corp. Released 2023. IBM SPSS Statistics for Macintosh, Armonk, NY: IBM Corp).

## Results

### Participant characteristics

**Fig. 1:**
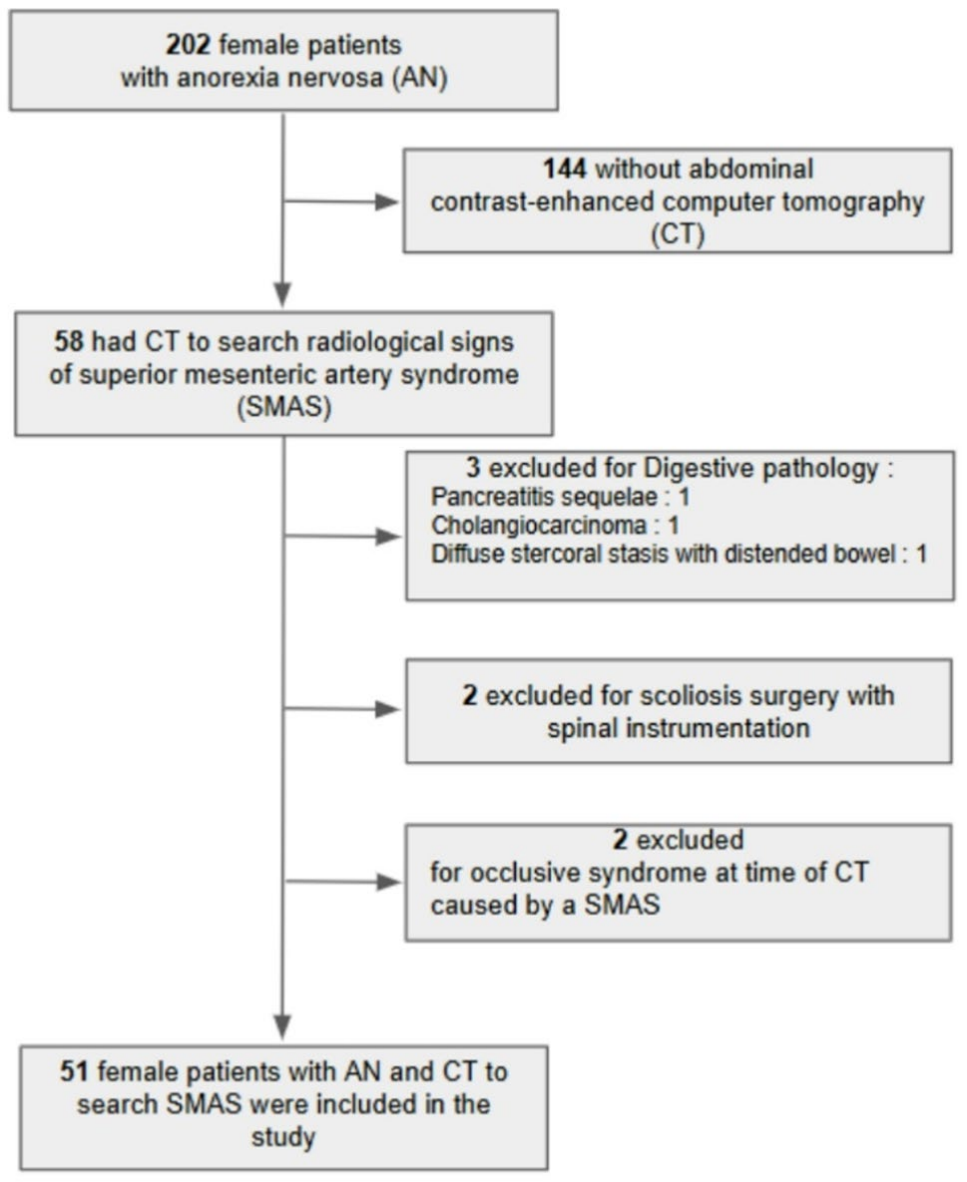
Flow chart

Among 202 consecutive female inpatients and outpatients attending the acute centre for nutrition diseases for AN, 58 underwent computed tomography (CT) to investigate for radiological signs of SMAS, based on clinical suspicion. Seven patients were excluded from the analyses: two presented with occlusive syndrome due to SMAS without prior digestive symptoms (one with AN-R, BMI 13.4 kg/m²; one with AN-B/P (vomiting), BMI 13.8 kg/m²) (Fig. 1).

Fifty-one patients diagnosed with AN who underwent CT imaging were included in the final analysis. Of these, 27 were inpatients and 24 were outpatients, and only seven (13.7%) were minors. Regarding anorexia nervosa subtypes, 30 patients had the restrictive type (AN-R) and 21 the binge-purge type (AN-B/P). Four patients presented associated comorbidities potentially responsible for digestive symptoms: three with Ehlers-Danlos syndrome, and one with irritable bowel syndrome.

The 51 selected patients exhibited characteristics similar to those of the 144 patients without CT scans, except for lower weight and BMI, and a higher frequency of digestive complaints. Participant characteristics are detailed in Table 1.

**Table 1:** Patients characteristics

	Patients with CT scan (*n* = 51)	Patients without CT scan (*n* = 144)	*P* value
Age (years), mean ± SD	27.7 ± 12.3	24.7 ± 11.2	0.11
Weight (kg), mean ± SD	39.8 ± 7.3	45 ± 8.8	**< 0.001**
Height (cm), mean ± SD	162.8 ± 6.5	163.4 ± 6.6	0.54
BMI (kg/m²), mean ± SD	14.9 ± 2.4	16.8 ± 3	**< 0.001**
minimal BMI (kg/m²), mean ± SD	13.7 ± 2.2	14.7 ± 2.7	**0.02**
maximal BMI (kg/m²), mean ± SD	22 ± 4.3	23.9 ± 6.5	0.06
Type of eating disorder, n (%)			0.21
AN-R	30 (58,8)	70 (48.6)	
AN-B/P	21 (41.2)	74 (51.4)	
Age of onset of eating disorder (years), mean ± SD	16.8 ± 3,8	18.4 ± 7	0.12
Patients presenting 1 or more digestive complaint at clinical evaluation, n (%)	48 (94.1%)	53 (36.8%)	**< 0.001**

*CT* computed tomography,* AN-R* AN-restricting subtype,* AN-B/P* AN-binge/purge subtype

### Analyses of the A-M space

CT scans analyses revealed a reduced A-M angle (< 25°) and/or distance (≤ 8 mm) in the majority of patients (Table 2). Forty-eight patients (94.1%) met radiological criteria for SMAS (angle < 25° or distance ≤ 8 mm). Duodenal dilation was present in 68.6% and left renal vein dilation in 39.2%. A-M distance was significantly but moderately correlated with angle (*r* = 0.41 [CI = 0.15–0.62], *p* < 0.003). The level of D3 varied, located between the second and fourth lumbar vertebrae and opposite the third vertebra in only 52.9% of patients, thus potentially modulating the relationship between A-M angle and distance. Nevertheless, the correlation coefficient between A-M angle and distance did not significantly differ when considering only the 27 patients with D3 located at the third lumbar vertebra level (*r* = 0.42, *p* < 0.03) and rates of duodenal dilation did not significantly differ according to lumbar level of D3 : 69.2% at L2, 66.7% at L3, 72.7% at L4 (*p* = 0.93).

**Table 2:** Radiological data and abdominal complaints of patients with CT scan

	Patients with CT (*n* = 51)
*Signs of SMAS*	
Aortomesenteric angle (°): mean ± SD/median [min - max]	15.8 ± 7.6/13 [8–39]
Aortomesenteric distance (mm): mean ± SD/median [min - max]	5.4 ± 1.9/5 [2–12]
Aortomesenteric angle < 25°, n (%)	44 (86.3%)
Aortomesenteric angle < 22°, n (%)	43 (84.3%)
Aortomesenteric distance ≤ 8 mm, n (%)	48 (94.1%)
Aortomesenteric distance ≤ 6 mm, n (%)	37 (72.5%)
Third duodenum stenosis, n (%)	42 (82.4%)
Duodenal dilation, n (%)	35 (68.6%)
*Third duodenum level*	
L2, n (%)	13 (25.5%)
L3, n (%)	27 (52.9%)
L4, n (%)	11 (21.6%)
*Signs of NS*	
Left renal vein stenosis, n (%)	18 (35.3%)
Renal vein dilation, n (%)	20 (39.2%)
Dilated ovarian vein, n (%)	13 (25.5%)
Pelvic varicose veins, n (%)	13 (25.5%)
*Other CT measurements*, mean ± SD	
Sagittal abdominal diameter (mm)	146 ± 13.8
Prevertebral sagittal diameter (mm)	61.9 ± 13.1
VAT (cm²)	13.6 ± 9.8
SAT (cm²)	33.8 ± 34.5
Skeletal Muscle Mass (cm²)	88.1 ± 19.2
Psoas Muscle Mass (cm²)	10.9 ± 3.1
Skeletal muscle mass index (cm²/m^2^)	33.2 ± 6.7
Psoas muscle mass index (cm²/m^2^)	4.1 ± 1.1
*Gastrointestinal symptoms*	
Dyspepsia/difficult to digest, n (%)	14 (27.5%)
Early satiety, n (%)	11 (21.6%)
Postprandial pain or discomfort, n (%)	41 (80.4%)
Nausea after meals, n (%)	17 (33.3%)
Spontaneous emesis after meals, n (%)	6 (11.8%)
Bloating, n (%)	27 (52.9%)
Eructation, n (%)	10 (19.6%)
Gastroesophageal reflux, n (%)	29 (56.9%)
Others symptoms, n (%)	17 (33.3%)
*Symptoms suggestive of NS*	4 (7.8%)

*CT* computed tomography,* SMAS* superior mesenteric artery syndrome,* NS* Nutcracker syndrome,* L* Lumbar vertebra,* VAT* visceral adipose tissue,* SAT* subcutaneous adipose tissue

### Relations between body composition and A-M measurements

BMI did not correlate significantly with A-M measurements (Supplementary Fig. 5S). No correlation was found between minimal BMI and A-M measurements. Notably, among three patients with AN/B-P, normal BMI, and digestive complaints (particularly gastroesophageal reflux), two had a A-M space reduction and duodenal dilation.

None of the following parameters-sagittal abdominal diameter, prevertebral sagittal diameter, or fat and muscle surfaces and their indices- were significantly associated with A-M distance.

### Digestive complaints and A-M space reduction

Postprandial pain/discomfort was the most frequent digestive complaint (80.4%) and spontaneous emesis after meals was present in 6 patients (11.8%). Symptoms suggestive of NS were reported in 4 patients (7.8%).

Digestive complaints did not differ significantly between patients with (*n* = 35) and without (*n* = 16) radiological signs of duodenal dilation, although gastroesophageal reflux approached statistical significance (65.7% vs. 37.5%, *p* = 0.06) (Table 3).

**Table 3:** Relationships between digestive complaints and duodenal dilation

	Patients with duodenal dilation (*n* = 35)	Patients without duodenal dilation (*n* = 16)	*P* value
*Gastrointestinal symptoms*			
Dyspepsia/difficult to digest, n (%)	9 (25.7%)	5 (31.3%)	0.68
Early satiety, n (%)	7 (20.0%)	4 (25.0%)	0.69
Postprandial pain or discomfort, n (%)	28 (80.0%)	13 (81.3%)	0.92
Nausea often after a meal, n (%)	13 (37.1%)	4 (25.0%)	0.59
Spontaneous emesis often after a meal, n (%)	5 (14.3%)	1 (6.3%)	0.72
Bloating, n (%)	19 (54.3%)	8 (50.0%)	0.78
Eructations, n (%)	8 (22.9%)	2 (12.5%)	0.39
Gastroesophageal reflux, n (%)	23 (65.7%)	6 (37.5%)	0.06
Other symptoms, n (%)	12 (34.3%)	5 (31.3%)	0.83

### Comparisons between AN subtypes (AN-R vs. AN-B/P)

Clinical and CT data comparisons between AN-R and AN-B/P patients are presented in Supplementary Table 1 S. BMI was lower in AN-R subgroup, but sagittal abdominal and prevertebral diameters and VAT did not differ significantly from those in the AN-B/P group. Skeletal muscle mass was lower in AN-R group, but proportional to BMI, as SMM/BMI was similar between groups. No difference was observed in CT data in A-M angle and distance, D3 stenosis, duodenal dilation, or NS signs.

Bloating was more common among patients with AN-R (66.7% vs. 33.3%, *p* = 0.03), while spontaneous postprandial emesis occurred exclusively in patients with AN-B/P (28.6% vs. 0%, *p* = 0.007). Notably, among the six AN/BP patients reporting spontaneous emesis, only four also engaged in self-induced vomiting. Clinical symptoms potentially attributable to NS were similarly distributed between the two groups. One AN-B/P patient with left lower quadrant pain exhibited CT findings consistent with left renal vein stenosis, renal vein dilatation, dilated ovarian vein and pelvic varicose veins. Another AN-B/P patient with pelvic pain showed no such CT abnormalities. In the AN-R group, one patient with left upper quadrant pain demonstrated CT evidence of left renal vein stenosis and renal vein dilatation only. A further AN-R patient, who reported left lateral lower back pain, and persistent headache (with normal MRI findings) showed no CT signs suggestive of NS.

### Impact of various A-M angles and distances

As detailed in supplementary Table 2 S, both an A-M angle < 25° and < 22° were equally predictive of impact on A-M space reduction, while an A-M distance ≤ 8 mm was most associated with this impact, being present in all but one patients with duodenal dilation and in all with signs of renal vein compression.

Combining A-M angle < 25° and A-M distance ≤ 8 mm was not contributible as all patients with an A-M angle < 25° also had an A-M distance ≤ 8 mm.

### Evolution after nutritional support

Ten patients (8 with AN-R and 2 with AN-B/P) underwent two CT scans, before and after nutritional support, with a mean interval of 367 ± 339 days between examinations. All presented with at least one gastrointestinal symptom at baseline, most commonly postprandial pain or discomfort (*n* = 10), bloating (*n* = 6), gastroesophageal reflux (*n* = 5), nausea after meals (*n* = 5); three patients reported symptoms suggestive of NS. No spontaneous postprandial emesis was observed.

Anthropometric and CT data changes in this subgroup are summarized in Table 4. Weight gain was primarily associated with increased subcutaneous adipose tissue and was accompanied by a significant increase in VAT, sagittal abdominal diameter, and prevertebral sagittal diameter.

Both A-M angle (+ 1.88° per 1 kg/m^2^ BMI increase) and A-M distance (+ 0.38 mm per 1 kg/m^2^ BMI increase) increased significantly (see Supplementary Fig. 6S). Of the seven patients with duodenal dilation before nutritional support, only two retained this abnormality after nutritional recovery. Left renal vein stenosis remained unchanged despite weight gain. Regarding symptom evolution, three patients experienced complete resolution of digestive complaints, one showed partial improvement (not difficulty to digest, but persistence of gastroesophageal reflux), five reported no change, and one was not reassessed for digestive complaints post-recovery.

**Table 4:** Comparison of anthropometric and CT data between initial assessment and reassessment after nutritional support

	First CT scan (*n* = 10)	Second CT scan (*n* = 10)	Evolution absolute relative	*P* value
Weight (kg)	39.7 ± 6.3	48.8 ± 5.9	9.1 ± 4.1 kg	**< 0.001**
			24.1 ± 11.8%	
BMI (kg/m²)	14.5 ± 2.1	17.8 ± 2.5	3.4 ± 1.6 kg/m²	**< 0.001**
			24.1 ± 11.8%	
A-M angle (°)	12.7 ± 2.6	19 ± 7.5	6.4 ± 5.9 °	**0.007**
			49.2 ± 42.0%	
A-M distance (mm)	4.9 +/1.4	6.2 ± 1.1	1.3 ± 1.7 mm	**0.03**
			43.0 ± 76.3%	
D3 stenosis	10	3	−7; −70%	0.24
Duodenal dilation	7	2	−5; −71.4%	**0.06**
Left renal vein stenosis, n (%)	3	3	0	0.34
Renal vein dilatation, n (%)	3	3	0	0.34
Dilated ovarian vein, n (%)	3	3	0	0.34
Pelvic varicose veins, n (%)	3	3	0	0.34
Sagittal abdominal diameter (mm)	146 ± 8.4	161.9 ± 13.5	15,3 ± 12.5 mm 10.5 ± 8.6%	**0.002**
Prevertebral sagittal diameter (mm)	62.4 ± 7.9	73.2 ± 12.2	10.8 ± 10.2 mm 17.7 ± 17.1%	**0.004**
VAT (cm²)	14.6 ± 4.7	33.7 ± 9.5	19.1 ± 8.8 cm² 151.6 ± 91.4%	**< 0.001**
SAT (cm²)	37.2 ± 37.5	110.6 ± 65.3	73.5 ± 37.1 cm² 527.9 ± 702.8%	**< 0.001**
Skeletal Muscle Mass (cm^2^)	87.9 ± 16.7	91.1 ± 18.8	3.1 ± 7.5 cm² 3.5 ± 9.4%	**< 0.001**
Psoas Muscle Mass (cm^2^)	11.6 ± 3.1	12.9 ± 3.3	1.3 ± 1.6 cm² 12 ± 15.8%	**< 0.001**
Skeletal muscle mass index (cm^2^/m^2^)	32.1 ± 5.9	33.3 ± 7.1	1.2 ± 2.8 3.5 ± 9.4%	**< 0.001**
Psoas muscle mass index (cm^2^/m^2^)	4.2 ± 1.1	4.7 ± 1.3	12 ± 15.8% 0.5 ± 0.6	**< 0.001**

Data are expressed as mean +/- SD or n (%)

*CT* computed tomography,* D3* third duodenum,* VAT* visceral adipose tissue,* SAT* subcutaneous adipose tissue

## Discussion

### Frequency of radiological features of SMA-syndrome in AN

This study focused on the A-M space reduction and its association with digestive complaints in patient with AN. Most published reports on superior mesenteric artery and NS are limited to case reports or small case series (maximum of 8 patients) [23]. To our knowledge, this is the largest series analyzing A-M space reduction in AN.

Of the 58 patients who underwent CT to investigate radiological signs of SMAS, three were excluded due to conccurent digestive pathology and two for occlusive syndrome at time of CT which was subsequently attributed to an undiagnosed SMAS. These findings underscore the importance of CT in excluding alternative causes of intestinal obstruction (e.g., tumors) and in diagnosing occlusive forms of SMAS, even in the absence of prior symptoms, as already reported by Ooi et al. [5]

The 51 patients included in the analysis nearly all presented with variable digestive complaints, most commonly postprandial abdominal pain and/or discomfort. A reduced A-M angle < 25° or A-M distance ≤ 8 mm usually considered as diagnosis criteria of SMAS [14] was observed in the majority of patients (94.1%), as well as repercussions on the duodenum with D3 stenosis and duodenal dilation in 82.4% and 68.6% respectively. No significant differences were found between AN-R and AN-B/P subgroups. These data reveal an unexpectedly high prevalence of radiological signs of SMAS (18.3%) among AN, since radiological signs of SMAS with duodenal dilation or occlusive syndrome was diagnosed in 37 patients among the total population of 202 AN patients followed in our center. This prevalence exceeds previous reports in dyspeptic patients [32] and in AN [23], but aligns with a recent study including AN patients [33]. Given that 36.8% of the 144 patients without CT scan also reported digestive complaints, the true prevalence of A-M space reduction in AN may exceed 20%, even if their mean BMI was higher than in the CT group.

### Influence of BMI and body composition on radiological findings of SMAS in AN

Notably, radiological signs of SMAS were identified in two patients with normal BMI, and no significant relationship was found between BMI and A-M angle or A-M distance. This contrasts with findings by Unal et al. who reported a significant correlation between BMI and A-M distance in a population of 50 women and 39 men with a normal mean BMI consecutively referred for CT investigation of the upper abdomen in a turkish university hospital (9 subjects having SMAS with duodenal dilation), and those of Ozkurt et al. [28] who found a moderate significant correlation between BMI and A-M angle and A-M distance in 524 subjects with various BMI referred for abdominal CT examinations due to different reasons (only 12 had low BMI and none had clinical signs of SMAS).

In our study, we also analyzed potential relationships between A-M data (angle and distance) and sagittal diameters, adipose tissue and muscle surfaces (absolute values and BMI-indexed ratios). No significant correlation was observed between A-M angle or distance and SAT, VAT, SMM, PMM, prevertebral sagittal diameter and sagittal abdominal diameter. This differs from previous reports of a significant positive relationship between VAT and both A-M angle and distance in children and adults with normal BMI, but those studies excluded patients with SMAS or clinical symptoms [34, 35].

### Radiological signs of NS in AN

Radiological signs of left renal vein entrapment and upstream dilation were frequent (39.2% of our population whose mean age was 27.7) and varicose pelvic veins were present in 65% of patients with left renal vein dilation. Although the co-existence of SMAS and NS has been reported in only a few case reports [20] 18 of the 20 patients with dilation of left renal vein, also exhibited duodenal dilation. Most patients with radiological signs of NS appeared to be free of symptoms suggestive of NS, but it cannot be excluded that abdominal discomfort attributed to SMAS may partly result from NS or that signs of pelvic congestion could be present during sexual activity [36]. Symptoms of pelvic venous congestion (e.g., chronic pelvic pain, dyspareunia, dysuria, and dysmenorrhea) were difficult to evaluate in our population due to confounding factors such as sexual trauma and functional hypothalamic amenorrhea. Furthermore, urine test for microscopic hematuria or proteinuria- hallmark manifestations of NS [21]- were not systematically performed in patients with radiological signs of NS.

### Radiological criteria of SMAS in AN

Our findings suggest that current radiological criteria for SMAS may be too stringent and require adaptation for population with low BMI, such as in AN.

While A-M angle and A-M distance are typically considered independent diagnostic criteria for SMAS, we observed a relatively weak relationship between these measures, possibly due to variations in the lumbar vertebra level of D3, which may alter A-M distance for a given angle. However, the correlation between A-M angle and A-M distance and duodenal dilation rates was not modulated by the lumbar vertebra level of D3. Other factors, such as a high fixation of duodenum by the ligament of Treitz [37] and a low origin of the superior mesenteric artery may also play a role, although our analysis of 5 patients at each lumbar vertebra level of D3 (L2, L3 and L4), revealed no differences in the origin level of this artery.

In this study, an A-M distance ≤ 8 mm was the most strongly associated with radiological signs of D3 stenosis and upstream dilation, left renal vein stenosis and upstream dilation, dilated ovarian vein and pelvic varicose veins. Coupling the A-M distance and the A-M angle (A-M distance ≤ 8 mm and/or A-M angle < 25°) did not provide additional predictive value, as this combination was present in all but one patient with duodenal dilation, and in all patients with signs of renal vein compression. These findings support the use of an 8 mm threshold for A-M distance in underweight patients and suggest that its presence should particularly alert the radiologist to look for signs of duodenal and left vein renal compression.

### Radiological features of SMAS and digestive complaints in AN

The presence of various digestive complaints was not strongly predictive of radiological features of SMAS, as these complaints did not significantly differ between patients with duodenal dilation (reflecting a slowdown in upstream intestinal transit) and those without. However, gastroesophageal reflux was nearly significant (*p*= 0.06). It is noteworthy that this symptom usually associated with excess of body weight, was more frequent (56.9%) in our sample than in the general population; a meta-analysis on the prevalence of GERD in subjects under 50 years reported a rate of 16.7% in women [38].

Moreover, the absence of digestive complaints does not exclude a SMAS as two patients in our cohort without previous digestive complaints presented with occlusive digestive syndrome due to SMAS at time of CT. This could be explained by a spontaneous decrease in food intake to prevent digestive complaints or by masking of symptoms due to the restriction and/or vomiting caracterizing the eating disorder.

Indeed, when comparing the two subtypes of anorexia nervosa, AN-R and AN-BP, bloating and early satiety were respectively two and three times less frequent in AN-B/P than in AN-R (*p* = 0.03 and 0.08).

Among the six AN/BP patients who reported spontaneous emesis, four also reported induced emesis, and no significant relationship could be found with duodenal dilation. This highlights the likely role of induced vomiting history on this digestive symptom rather than that of a gastric distension, which has been recently reported as frequent in both AN-R and AN-B/P [8].

#### Recovery after nutritional support

Changes in CT aortomesenteric data after nutritional support were analyzed for the first time in ten patients, (eight with AN-R and two with AN-B/P), all of whom had digestive complaints. There was a significant increase in A-M angle (+ 1.88° per 1 kg/m^2^ BMI increase) and A-M distance (+ 0.38 mm per 1 kg/m^2^ BMI increase). The main change of radiological signs of SMAS between patients with regression of symptoms (total = 3 and partial = 1) and patients with persistence of digestive complaints were A-M distance. Weight gain was primarily due to changes in SAT and was also accompanied by significant increase in VAT, sagittal abdominal diameter, and prevertebral sagittal diameter. Of the seven patients with duodenal dilation before nutritional support, only two still exhibited this abnormality after nutritional recovery (Table 4). In contrast, left renal vein stenosis remained unchanged, suggesting that varicose veins are less reversible than digestive consequences.

In the literature, there is no consensus regarding the minimal weight gain or BMI required to alleviates SMAS symptoms. Weight gain between the two CT scans was 24.1 ± 11.8% (*p*< 0.001) and led to regression of digestive complaints limiting food consumption in four of the nine patients assessed for symptoms, thus supporting the attribution of these symptoms to a SMAS. The persistence of digestive symptoms in the remaining patients could be explained by gastric motor disorders of other origins or visceral hypersensitivity [39].

The challenge for the future will be to determine in a larger population, the minimum VAT area (at a specified lumbar level) necessary to prevent or treat a SMAS.

### Limitations of the study

As a retrospective study, some digestive complaints may have not been identified in hospitalization and medical consultation records, and their intensity could not be determined. Given the potentially multifactorial nature of digestive disorders in AN, some symptoms reported by patients may have been falsely attributed to radiological features of SMAS, thereby reducing the ability to detect significant relationships between digestive complaints and A-M CT data. These elements explain why we were unable to give the real prevalence of SMAS in our sample, and beyond this consideration it highlights the difficulty to confirm SMAS diagnosis in AN.

Another limitation is that CT scans were reanalyzed by a single radiologist, and there is currently no literature on an anthropometric definition of duodenal dilation.

## Conclusion

Radiological signs of SMAS are frequent in patients with AN, particularly in those with digestive or abdominal complaints. Gastroesophageal reflux may serve as a possible predictor of duodenal dilation in this population, although this requires confirmation in a prospective study with a larger sample. Radiological signs of NS are also frequently associated to A-M space reduction, but their clinical impact remains to be clarified.

This study highlights that the CT criterion most significantly associated with the consequences of A-M space reduction is an A-M distance ≤ 8 mm, and that BMI is not significantly correlated to A-M angle and A-M distance in patients with AN. It also confirms that a nutritional support alleviates digestive complaints by increasing A-M angle and A-M distance, though not in all patients. A prospective study is needed to better define the diagnostic criteria of SMAS and NS in AN patients.

## Supplementary Information

Supplementary Material 1

## Data Availability

The data that support the findings of this study are available on request from the corresponding author. The data are not publicly available due to privacy or ethical restrictions.

## Abbreviations

AN: Anorexia nervosa
AN-B/P: Anorexia nervosa of the binge-purge type (AN-B/P)
AN-R: Anorexia nervosa restrictive type
A-M: Aorto-mesenteric
BMI: Body mass index
CT: Computerized tomography
D3: Third part of the duodenum
GERD: Gastroesophageal reflux diseases
NS: Nutcracker syndrome
PMM: Psoas muscle mass
SAT: Subcutaneous adipose tissue
SD: Standard deviation
SMA: Superior mesenteric artery
SMAS: Superior mesenteric artery syndrome
SMM: Skeletal muscle mass
VAT: Visceral adipose tissue
