# Supplementary material for: Aorto-mesenteric space reduction in women with anorexia nervosa: retrospective audit and analysis
Source: J Eat Disord. 2026 Mar 6;14:90. doi: 10.1186/s40337-026-01566-1 (PMC13094017; doi:10.1186/s40337-026-01566-1)
Supplement: Supplementary file 1 — Supplementary Material 1 [file 40337_2026_1566_MOESM1_ESM.docx]

**Figure 1S : Nutcracker phenomenon and varicocele**

1. Compression of LRV between superior mesenteric artery and aorta and Left renal vein dilation upstream


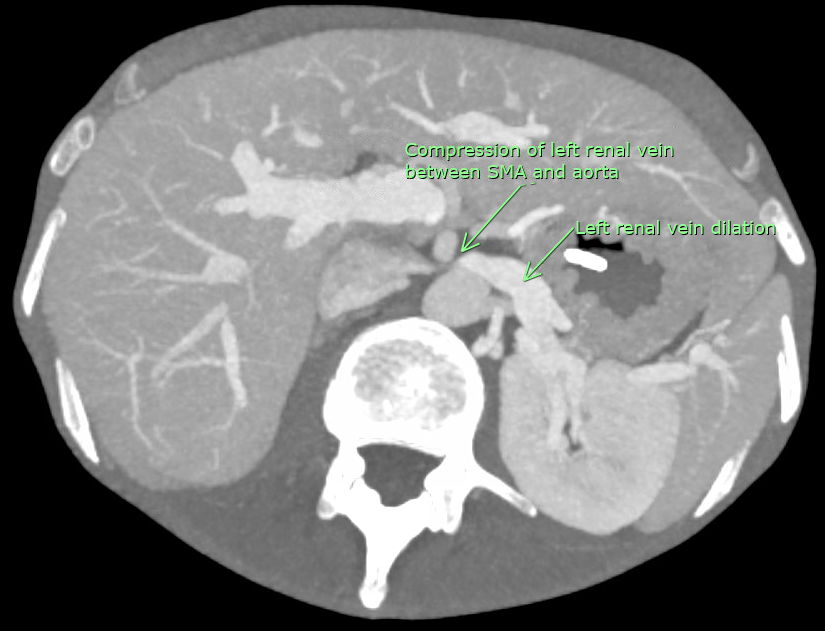


1. Left ovarian vein dilation


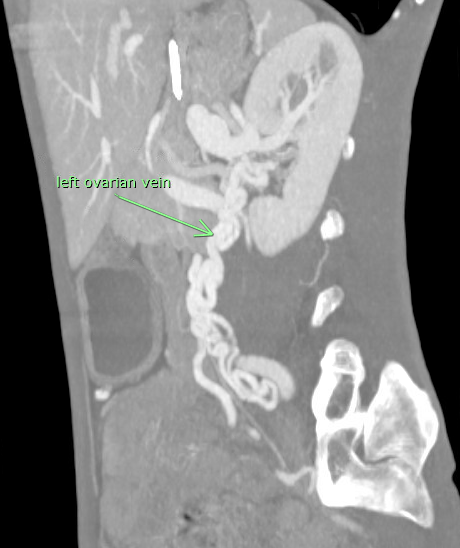


1. Pelvic varicose veins


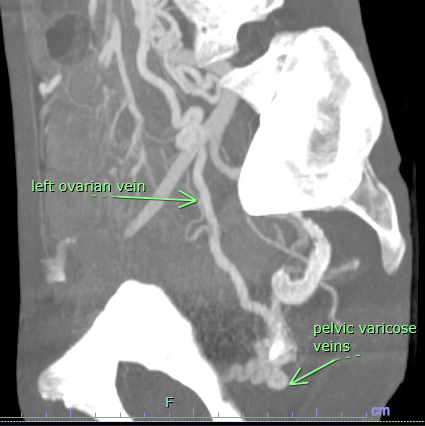


**Figure 2S : Aortomesenteric measurement**


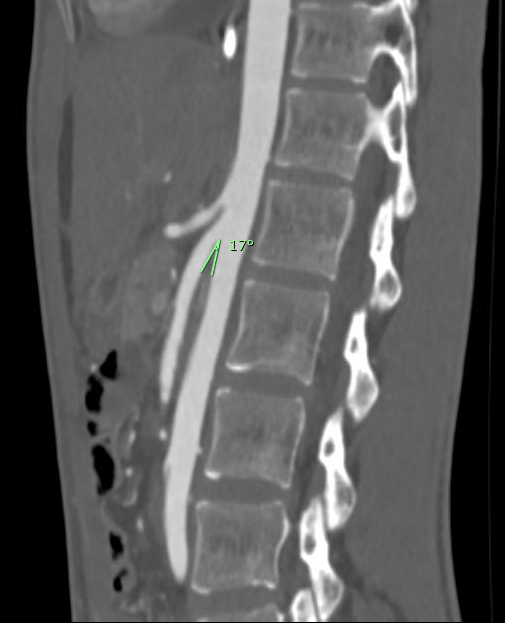

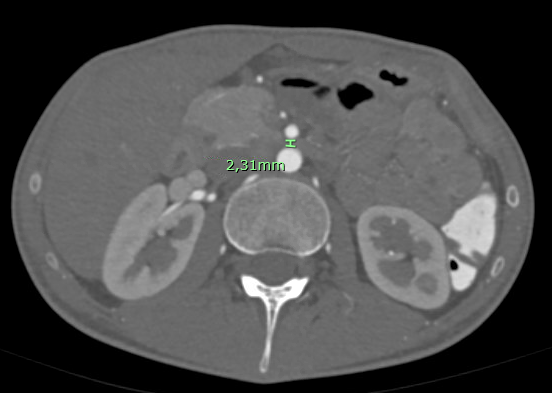


**Figure 3S :**

**Semi automatical result of VAT and SAT (cm²) (L4 level)**


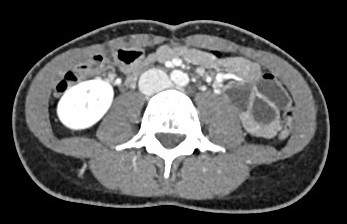

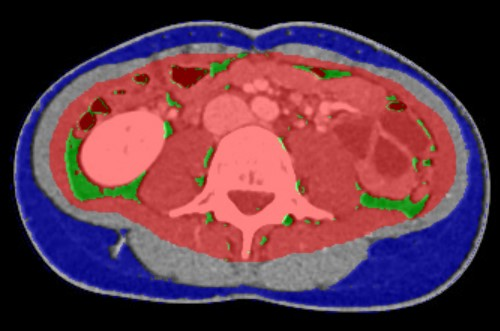


Green :Visceral adipose tissue (VAT). Blue : Subcutaneous adipose tissue (SAT)

**Semi-automatical result of SMM (cm2) (L3 level)**


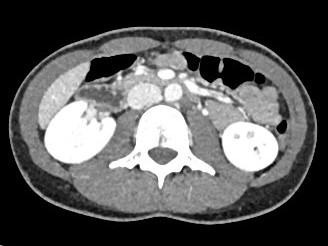

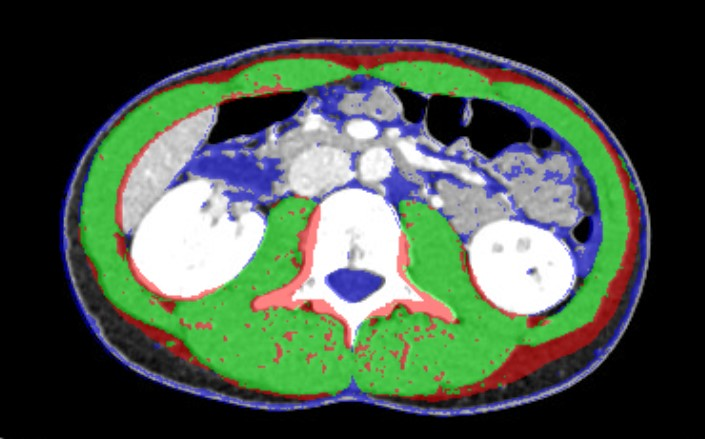


Green: Visceral adipose tissue (VAT)

**Semi-automatical result of PMM (cm2) (L3 level)**


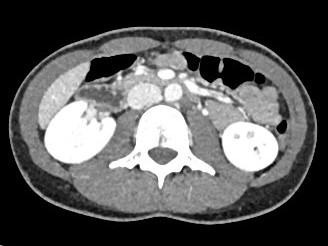

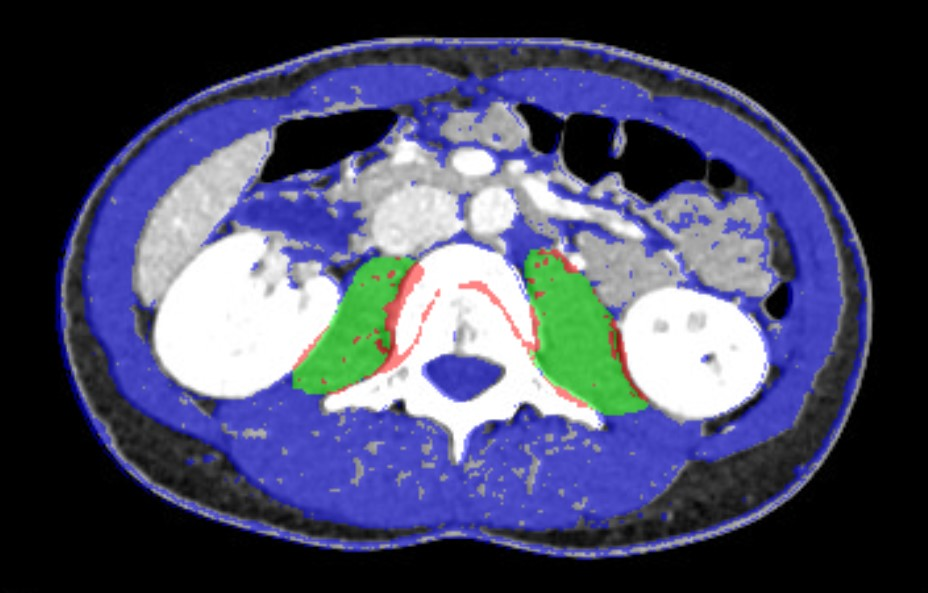


Green : Psoas muscle mass (PMM)

**Figure 4S : Sagittal abdominal diameter and Prevertebral sagittal diameter (L3 level)**


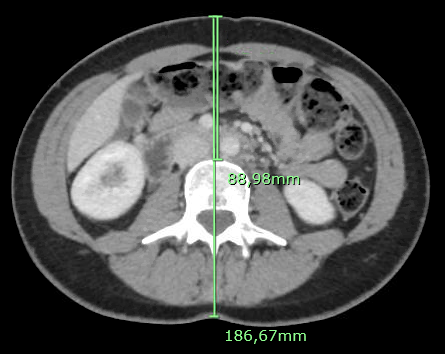


**Table 1S. Comparison between 2 subgroups of AN patients (AN-R vs AN-B/P) with CT measurements**

|  | **AN-B/P**  (n=21) | **AN-R**  (n=30) | *P value* |
| --- | --- | --- | --- |
| Age (years), mean ± SD | 30.6 ± 12.4 | 25.8 ± 12.1 | 0.17 |
| Weight (kg), mean ± SD | 43.0 ± 7.6 | 37.6 ± 6.3 | 0.07 |
| Height (cm), mean ± SD | 162 ± 6.6 | 163 ± 6.5 | 0.68 |
| BMI (kg/m²), mean ± SD | 16.3 ± 2.5 | 14.1 ± 1.9 | **<0.001** |
| Minimal BMI (kg/m²), mean ± SD | 14.8 ± 2.3 | 12.9 ± 1.9 | **0.02** |
| Maximal BMI (kg/m²), mean ± SD | 23.1 ± 3.9 | 21.3 ± 4.5 | 0.14 |
| Age of onset of eating disorder (y), mean ± SD | 16.3 ± 3.8 | 17.1 ± 3.9 | 0.45 |
| *Signs of SMAS*  Aortomesenteric angle (°), mean ± SD  Aortomesenteric distance (mm), mean ± SD  Aortomesenteric angle < 25°  or distance ≤ 8 mm, n (%)  Aortomesenteric angle < 25°, n (%)  Aortomesenteric angle < 20°, n (%)  Aortomesenteric distance ≤ 8 mm, n (%)  Aortomesenteric distance ≤ 6 mm, n (%)  Third duodenum stenosis, n (%)  Duodenal dilation, n (%) | 16.9 ± 9.1  5.6 ± 2.2  19 (90.5%)  17 (80.9%)  16 (76.2%)  19 (90.5%)  14 (66.7%)  19 (90.5%)  17 (80.9%) | 15.1 ± 6.3  5.3 ± 1.7  29 (96.7%)  28 (93.3%)  26 (86.7%)  29 (96.7%)  23 (76.7%)  23 (76.6%)  18 (60.0%) | 0.41  0.50  0.75  0.36  0.55  0.75  0.43  0.20  0.11 |
| *Signs of NS*  Left renal vein stenosis, n (%)  Renal vein dilation, n (%)  Dilated ovarian vein, n (%)  Pelvic varicose veins, n (%) | 2 (9.5%)  8 (38.1%)  10 (47.6%)  6 (28.6%) | 2 (6.7%)  10 (33.3%)  10 (33.3%)  7 (23.3%) | 0.71  0.73  0.30  0.67 |
| *Other CT measurements*, mean ± SD  Sagittal abdominal diameter (mm)  Prevertebral sagittal diameter (mm)  VAT (cm²)  SAT (cm²)  Skeletal Muscle Mass (cm²)  Psoas Muscle Mass (cm²)  Skeletal muscle mass index (cm²/m^2^)  Psoas muscle mass index (cm²/m^2^)  SMM/BMI  PMM/BMI | 148.9 ± 16.4  63.1 ± 16.1  15.4 ± 9.9  45.56 ± 38.8  97.1 ± 21.7  12.2 ± 2.9  36.8 ± 7.5  4.6 ± 1.1  5.97 ± 0.9  0.75 ± 0.2 | 143.8 ± 11.5  61.0 ± 10.8  12.4 ± 9.8  26.1 ± 29.5  81.7 ± 14.4  10.0 ± 2.8  30.7 ± 4.8  3.8 ± 1.1  5.82 ± 0.7  0.71 ± 0.2 | 0.59  0.19  0.31  **0.05**  **0.04**  **0.009**  **<0.001**  **0.005**  0.52  0.36 |
| *Gastrointestinal symptoms*  Dyspepsia/ difficult to digest, n (%)  Early satiety, n (%)  Postprandial pain or discomfort, n (%)  Nausea often after a meal, n (%)  Spontaneous emesis often after a meal, n (%)  Bloating, n (%)  Eructation, n (%)  Gastroesophageal reflux (and pyrosis), n (%)  Other symptoms, n (%) | 4 (19.1%)  2 (9.5%)  16 (76.2%)  6 (28.6%)  6 (28.6%)  7 (33.3%)  4 (19.0%)  14 (66.7%)  4 (19.0%) | 10 (33.3%)  9 (30%)  25 (83.3%)  11 (36.6%)  0 (0%)  20 (66.7%)  6 (20%)  15 (50%)  13 (43.3%) | 0.21  0.08  0.72  0.76  **0.007**  **0.03**  0.93  0.37  0.13 |
| *Symptoms suggestive of NS,* n (%) | 2 (9.5%) | 2 (6.7%) | 0.71 |

*AN-R: AN‐restricting subtype, AN-B/P: AN‐ binge/purge subtype, CT = computed tomography, SMAS = Superior Mesenteric Artery Syndrome, NS = Nutcracker Syndrome, VAT = Visceral Adipose Tissue, SAT = Subcutaneous Adipose Tissue, SMM = Skeletal Muscle Mass, PMM = Psoas Muscle Mass*

**Table 2S. Impact on the duodenal and venous structures according to aorto-mesenteric angle and distance values**

|  | **D3 stenosis**    (n = 42) | **Duodenal dilation**    (n = 35) | **Left renal vein stenosis**  (n = 18) | **Renal vein dilation**    (n = 20) | **Dilated ovarian vein**    (n = 13) | **Pelvic varicose veins**    (n = 13) |
| --- | --- | --- | --- | --- | --- | --- |
| Angle < 25°  (n = 44) | 40 (95.2%) | 33 (94.3%) | 17 (94.4%) | 19 (95.0%) | 12 (92.3%) | 12 (92.3%) |
| Angle < 22°  (n = 43) | 40 (95.2%) | 33 (94.3%) | 17 (94.4%) | 19 (95.0%) | 12 (92.3%) | 12 (92.3%) |
| Angle < 20°  (n = 42) | 39 (92.9%) | 33 (94.3%) | 17 (94.4%) | 18 (90.0%) | 12 (92.3%) | 12 (92.3%) |
| Angle < 16°  (n = 33) | 33 (78.6%) | 27 (77.1%) | 16 (88.9%) | 17 (85.0%) | 11 (84.6%) | 11 (84.6%) |
| Distance ≤ 8mm  (n = 48) | 41 (97.6%) | 34 (97.1%) | 18 (100%) | 20 (100%) | 13 (100%) | 13 (100%) |
| Distance ≤ 7 mm  (n = 45) | 38 (90.5%) | 31 (88.6%) | 15 (83.3%) | 18 (90.0%) | 12 (92.3%) | 11 (84.6%) |
| Distance ≤ 6 mm  (n = 37) | 32 (76.2%) | 27 (77.1%) | 13 (72.2%) | 14 (70.0%) | 11 (84.6%) | 10 (76.9%) |
| Angle < 25° or distance ≤ 8 mm  (n = 47) | 40 (95.2%) | 33 (94.3%) | 17 (94.4%) | 19 (95.0%) | 12 (92.3%) | 12 (92.3%) |

*D3 = third duodenum, data in parentheses are percentages of patients relating to the data in columns*

**Figure 5S : Correlation between BMI and aorto-mesenteric measurements**


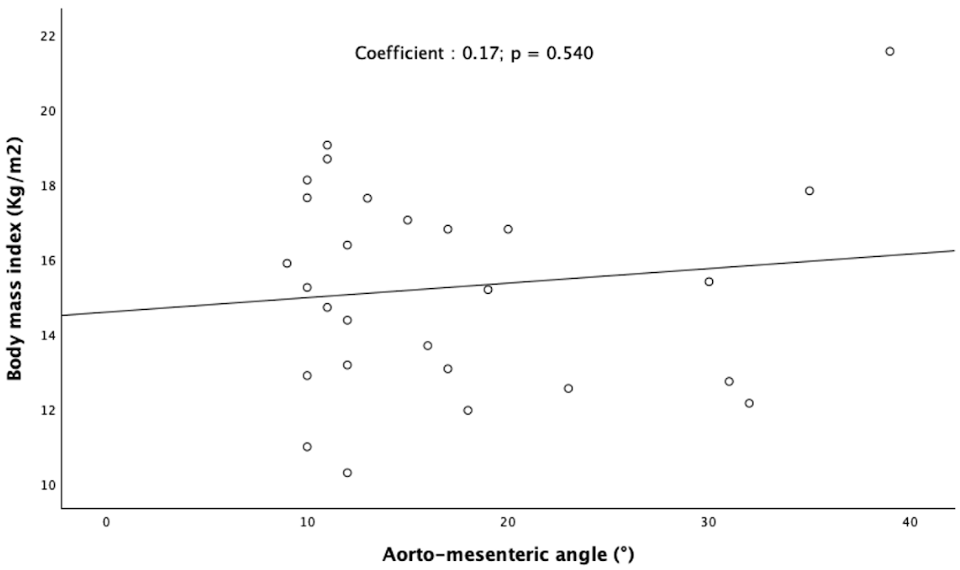

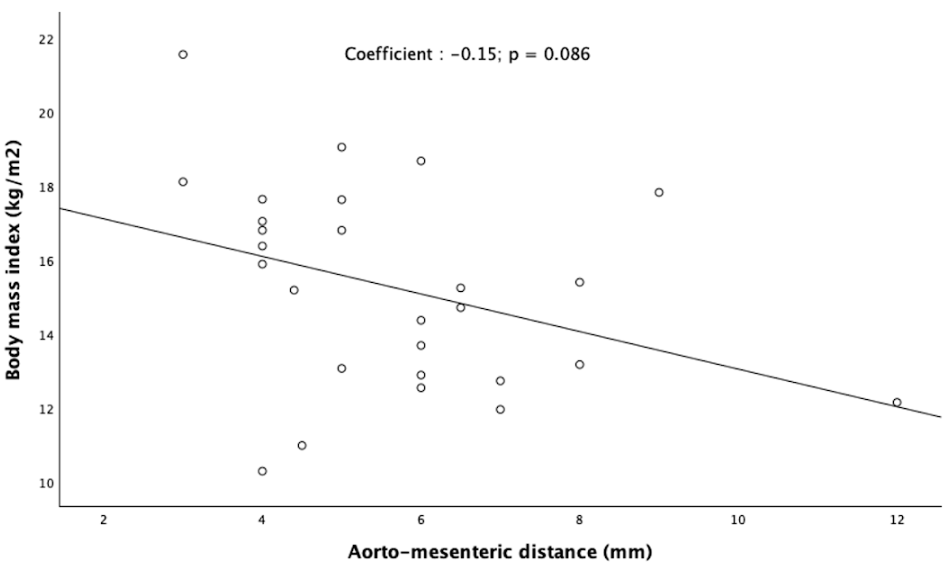


**Figure 6S : Comparison between CT at initial assessment and after nutrition support**

Aortomesenteric measurement at initial assessment and **after nutrition support**

| 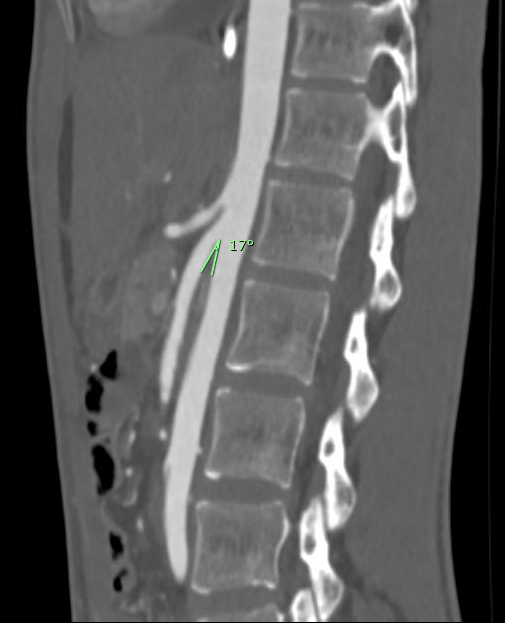 | 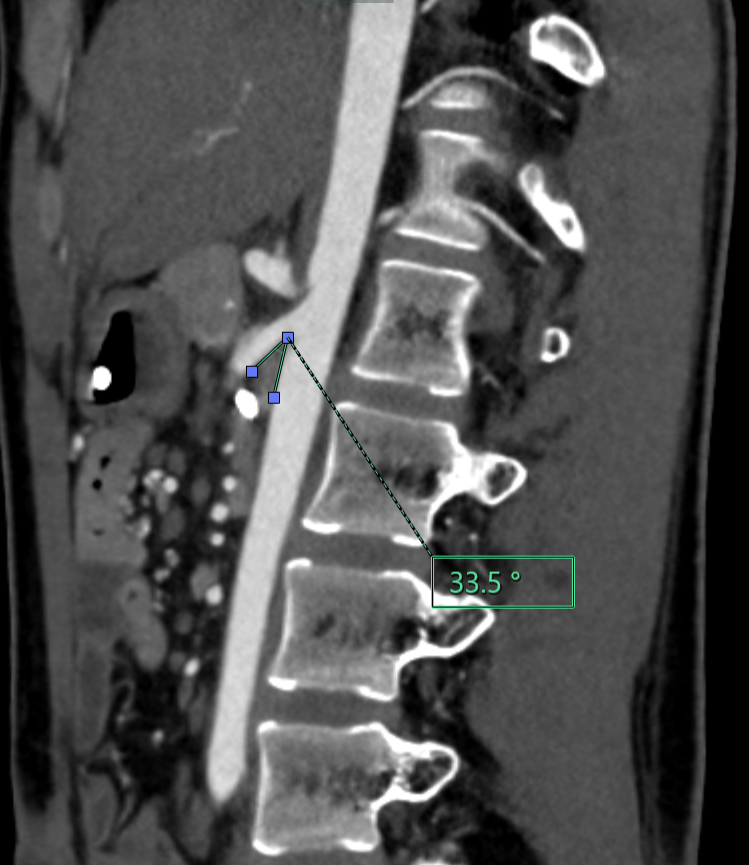 |
| --- | --- |
| 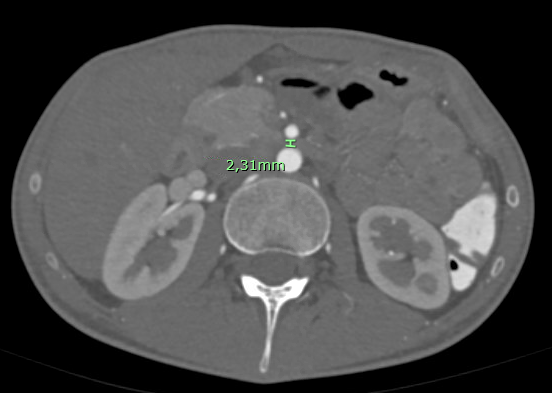 | 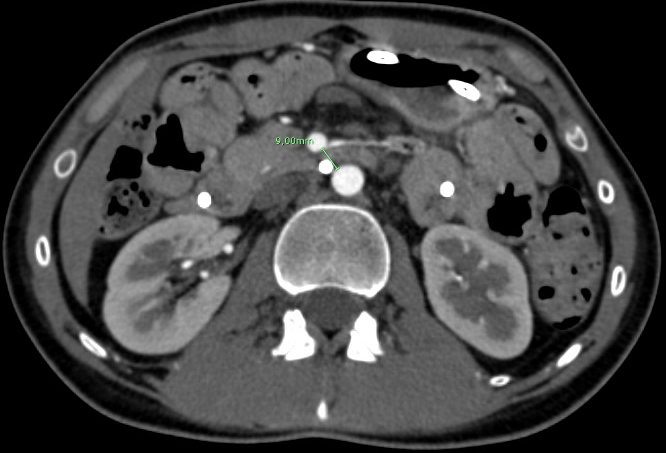 |
| Before weight restoration (body mass index, 13.8 kg/m2) *aorto-SMA angle : 17°, Aorto-SMA distance : 2.3 mm* | After weight restoration (body mass index, 17.3 kg/m2) *aorto-SMA angle : 33.5°, Aorto-SMA distance : 9 mm* |
